# MELD-XI score predict no-reflow phenomenon and short-term mortality in patient with ST-segment elevation myocardial infarction undergoing primary percutaneous coronary intervention

**DOI:** 10.1186/s12872-022-02556-2

**Published:** 2022-03-18

**Authors:** Xin-Tao Zhang, Zhao-Rong Lin, Lin Zhang, Zi-Wen Zhao, Liang-Long Chen

**Affiliations:** 1 Department of Cardiology, Union Hospital, Fujian Medical University, 29 Xin-Quan Road, Fuzhou, 350001 Fujian People’s Republic of China; 2 Fujian Institute of Coronary Artery Disease, Fuzhou, 350001 Fujian People’s Republic of China

**Keywords:** No-reflow phenomenon, MELD-XI, Short-term outcome, ST-segment elevation myocardial infarction, Liver and renal dysfunction, Percutaneous coronary intervention

## Abstract

**Introduction:**

No-reflow phenomenon (NRP) is one of the complications that mostly occur during percutaneous coronary intervention (PCI). In this study, we comprehensively examined the relationship between the model for end-stage liver disease-XI (MELD-XI) score and NRP. Moreover, we discussed whether the MELD-XI score could be considered as an accurate risk assessment score of patients with ST-segment elevation myocardial infarction (STEMI) who are candidates for PCI.

**Methods:**

This retrospective study involved 693 patients with acute STEMI and who underwent an emergency PCI. They were divided into a normal reflow group or a no-reflow group on the basis of the flow rate of post-interventional thrombolysis in myocardial infarction. Univariate, multivariate logistic regression, and Cox regression analyses were performed to identify the independent predictors of NRP in both groups. Receiver operator characteristic (ROC) curves and Kaplan–Meier curves were plotted to estimate the predictive values of the MELD-XI score.

**Results:**

MELD-XI score was found to be an independent indicator of NRP (odds ratio: 1.247, 95% CI: 1.144–1.360, *P* < 0.001). Multivariate Cox regression analysis also revealed that the MELD-XI score is an independent prognostic factor for 30-day all-cause mortality (hazard ratio: 1.155, 95% CI: 1.077–1.239, *P* < 0.001). Moreover, according to the ROC curves, the cutoff value of the MELD-XI score to predict NRP was 9.47 (area under ROC curve: 0.739, *P* < 0.001). The Kaplan–Meier curves for 30-day all-cause mortality revealed lower survival rate in the group with a MELD-XI score of > 9.78 (*P* < 0.001).

**Conclusion:**

The MELD-XI score can be used to predict NRP and the 30-day prognosis in patients with STEMI who are candidates for primary PCI. It could be adopted as an inexpensive and a readily available tool for risk stratification.

## Introduction

Primary percutaneous coronary intervention (PPCI) can greatly reduce the mortality and disability rate of patients with acute ST-segment elevation myocardial infarction (STEMI). However, no-reflow phenomenon (NRP) still frequently occurs in the treatment of patients with STEMI via PCI. NRP is an important predictor of poor short-term and long-term adverse outcomes for patients who have undergone PCI [1]. Owing to the importance of NRP, a fast and an effective method for stratifying the risk of patients with acute STEMI who are candidates to undergo PPCI.

The model for end-stage liver disease-XI score (MELD-XI, excluding international normalized ratio) was originally used to evaluate derangements of liver and renal functions. Furthermore, the MELD-XI score has been found to be associated with poor prognosis of heart failure [2]. Renal function status is independently correlated with the mortality rate of elderly patients with STEMI who had undergone PCI [3]. Bilirubin levels are not only independently related to no-reflow during hospitalization but also in-hospital major adverse clinical events (MACEs) [4]. Elevated serum creatinine levels upon admission are also one of the risk factors for NRP [5, 6].

In this study, we aimed to assess whether the MELD-XI score calculated upon admission can accurately predict NRP and 30-day prognosis in patients with STEMI who will undergo PPCI.

## Methods

### Study population

In this study, we retrospectively enrolled 693 patients presenting with STEMI admitted to our emergency cardiovascular department appropriate for PPCI from January 2018 to July 2021. Infarct-related artery blood flow was evaluated on the basis of the flow grade of thrombolysis in the myocardial infarction (TIMI). According to the post-PCI TIMI flow grade, the study population was further subdivided into a no-reflow group (TIMI flow grade of ≤ 2) and a normal reflow group (TIMI flow grade of > 2).

The TIMI flow grades were defined as follows: Grade 0 referred to the lack of antegrade blood flow through the vessel as determined by angiography after complete vascular occlusion. In Grade 1, the contrast agent was not completely blocked, and a small amount of the contrast material could flow through the occlusion site but could not fill the distal coronary artery. In Grade 2, the contrast agent filled the distal coronary vessels, but blood flow was slower than that in Grade 3. Grade 3 denoted normal coronary blood flow.

The diagnostic criteria for acute STEMI adopted in this study were as follows. First, the patients presented with typical acute chest pain that lasted for > 20 min. Second the patients had at least two contiguous leads with ST-segment elevation of 2.5 mm in men aged < 40 years, 2 mm in men aged > 40 years, or 1.5 mm in women in leads V2–V3 and/or 1 mm in the other leads, or had newly presented left bundle branch block [7].

The exclusion criteria were as follows: (1) patients who had previous acute thrombolytic therapy before coming to our emergency department; (2) patients who rejected percutaneous stent implantation; (3) patients with end-stage liver cirrhosis or severe kidney disease and undergoing dialysis; (4) presence of infectious or chronic inflammatory-autoimmune disease or malignancy.

### Coronary angiography and procedure

All patients were prescribed and administered via the radial artery with load-dose drugs of aspirin (300 mg), ticagrelor (180 mg), or clopidogrel (600 mg) before PPCI. In all patients, coronary angiography was performed following standard techniques through the radial approach. The various parameters and methods of the operation were determined by the individual operators.

The results of angiogram and post-PCI TIMI flow grade were independently analyzed in a double-blind manner by two interventional cardiologists. NRP was defined as a coronary TIMI grade flow of ≤ 2 after stent implantation [8, 9].

### Measurement, data collection, and endpoint

Fasting blood samples were obtained from all subjects prior to coronary angiography. Serum high-sensitivity C-reactive protein (hs-CRP), total cholesterol (TC), high-density lipoprotein cholesterol (HDL-C), low-density lipoprotein cholesterol, triglycerides, uric acid, serum albumin, creatine kinase-MB (CK-MB), N-terminal pro B-type natriuretic peptide, serum creatine (sCr), and serum total bilirubin (TB) levels were measured. Left ventricular ejection fraction (LVEF) was obtained from the clinical echocardiographic report prior to coronary angiogram.

Two researchers collected the relevant demographic and clinical characteristics of all patients from their hospital files and electronic medical records and checked for errors. Another researcher collated the information and analyzed the data. The MELD-XI score was calculated as follows: 5.11 × (ln total bilirubin, mg/dL) + 11.76 × (ln creatinine, mg/dL) + 9.44 [10].

The primary endpoint was 30-day all-cause mortality. The discharged patient were followed up via telephone interviews. The research protocol was approved by the Ethics Committee of the Union Hospital affiliated to Fujian Medical University. All procedures were conducted in accordance with the Declaration of Helsinki (as revised in 2013).

### Statistical analysis

Whether the distribution of continuous variables was normal was evaluated via the Kolmogorov–Smirnov test. Continuous data with a normal distribution were expressed as mean ± standard deviation. The median and interquartile ranges were used to describe continuous variables with a skewed distribution. Continuous variables were compared using an unpaired Student’s t-test or Mann–Whitney U test. Categorical variables were presented as frequency and compared using Chi-square test or Fisher exact test.

The potential predictors of NRP and 30-day mortality were determined via univariate and multivariate analyses. The area under the receiver operator characteristic (ROC) curve (AUC) was plotted to evaluate the predictive value of the MELD-XI scores. Two-tailed tests were applied in all statistical tests, and *P* < 0.05 was considered statistically significant. The independent predictors of the primary endpoint was analyzed through the multivariate Cox proportional hazards regression model. On the basis of the results of univariate regression analysis, age, heart failure, hypertension, LVEF, hs-CRP, CK-MB, and the MELD-XI scores were entered into the Cox multivariate regression model. Kaplan–Meier curves were plotted to analyze the 30-day survival, and statistical differences between the two groups were assessed using the log rank test. All data collected were statistically analyzed using SPSS 25.0 software (SPSS Inc., IBM Corporation, Armonk, New York, USA).

## Results

The study population’s demographic, clinical, laboratory, and procedural characteristics are listed in Table 1. The overall study population was divided into two groups: a normal reflow group (n = 547; 457 men with a mean age of 62.34 ± 12.46 years) and a no-reflow group (n = 146; 117 men with a mean age 63.95 ± 12.38 years). The incidence of NRP among the patients with STEMI was 21.0%. Notable differences were observed in their demographic and laboratory parameters, including heart failure, peripheral vascular history, hypertension, diabetes mellitus, SBP, HDL-C, BUN, sCr, hs-CRP, and TB levels (Table 1). In addition, the MELD-XI scores in the normal reflow group were substantially lower than those in the no-reflow group. However, no remarkable differences between the groups were observed in terms of gender, age, hyperlipidemia, smoking status, LVEF, anterior myocardial infarction, time to hospital of > 4 h, and multiple stenosis vessels with regard to baseline.

**Table 1:** Baseline characteristics of the study participants

Variable	Normal (n = 547)	No-reflow (n = 146)	*P* value
Clinic and demographic characteristics			
Age (years)	62.34 ± 12.46	63.95 ± 12.38	0.181
Male, n (%)	457 (83.5%)	117 (80.1%)	0.326
Smoking, n (%)	369 (67.5%)	86 (58.9%)	0.053
Heart failure (Killip II–IV, class), n (%)	136 (24.9%)	54 (37.0%)	0.004
Hypertension, n (%)	306 (55.9%)	103 (70.5%)	0.001
Diabetes mellitus, n (%)	168 (30.7%)	73 (50.0%)	< 0.001
Stroke or TIA, n (%)	53 (9.7%)	15 (10.3%)	0.876
Family history, n (%)	22 (4.0%)	6 (4.1%)	0.962
Peripheral vascular history, n (%)	11 (2.0%)	14 (9.6%)	< 0.001
Atrial fibrillation, n (%)	34 (6.2%)	9 (6.2%)	0.996
Hyperlipidaemia, n (%)	329 (60.1%)	77 (52.7%)	0.106
Heart rate (bpm)	78.00 ± 15.11	78.46 ± 15.70	0.699
SBP, mmHg	125.76 ± 22.37	130.47 ± 24.82	0.042
DBP, mmHg	78.04 ± 13.72	79.86 ± 16.03	0.202
LVEF (%)	50.66 ± 9.56	49.71 ± 9.51	0.327
Anterior myocardial infarction, n (%)	310 (56.7%)	81 (55.5%)	0.796
Time to hospital > 4 h	382 (69.8%)	100 (68.5%)	0.754
Laboratory parameters			
TC (mmol/L)	4.55 ± 1.07	4.63 ± 1.06	0.557
TG (mmol/L)	1.44 (1.05–2.08)	1.39 (1.02–2.05)	0.056
LDL-C (mmol/L)	3.03 (2.41–3.69)	3.08 (2.51–3.72)	0.623
HDL-C (mmol/L)	0.96 (0.82–1.15)	1.05 (0.90–1.24)	0.001
BUN (mmol/L)	4.90 (4.00–6.30)	5.75 (4.43–7.48)	< 0.001
sCr (mg/dL)	0.83 (0.74–0.97)	0.97 (0.81–1.19)	< 0.001
hs‐CRP (mg/L)	8.13 (3.19–22.60)	12.10 (4.57–32.53)	0.017
CK-MB (U/L)	88.85 (28.53–211.38)	82.85 (30.35–201.325)	0.789
UA (μmol/L)	367.19 ± 108.31	374.88 ± 111.34	0.465
HCY (μmol/L)	9.19 (7.37–11.24)	9.89 (7.72–12.09)	0.059
TB (mg/dL)	0.72 (0.54–0.93)	0.91 (0.59–1.19)	< 0.001
Nt-proBNP (pg/ml)	629.50 (181.75–1542.75)	807.50 (181.25–2104.75)	0.056
MELD-XI score	9.44 (9.44–10.29)	10.67 (9.65–12.59)	< 0.001
Preprocedural characteristics			
Multiple stenosis vessels, n (%)	345 (63.1%)	96 (65.8%)	0.549
LMCA, n (%)	36 (6.6%)	7 (4.8%)	0.563

*SBP* systolic blood pressure, *DBP* diastolic blood pressure, *LVEF* left ventricular ejection fraction, *TC* total cholesterol, *TG* triglycerides, *LDL-C* low density lipoprotein cholesterol, *HDL-C* high density lipoprotein cholesterol, *BUN* blood urea nitrogen, *sCr* serum creatine, *hs-CRP* high sensitivity C-reactive protein, *CK-MB* creatine kinase-MB, *UA* uric acid, *HCY* homocysteine, *TB* total bilirubin, *Nt-proBNP* N-terminal pro-brain natriuretic peptide, *LMCA* left main coronary artery

**Fig. 1:**
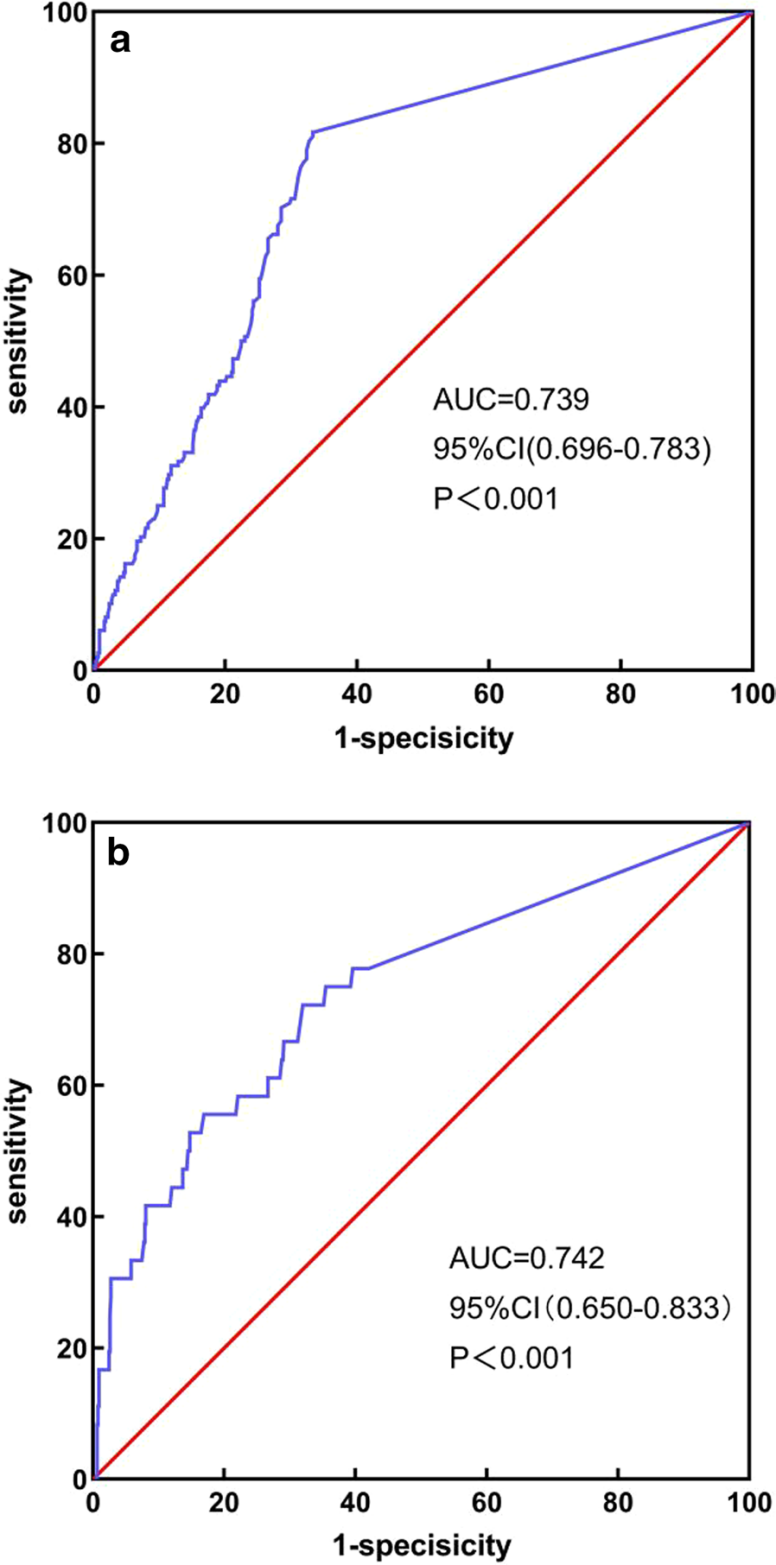
**a** ROC curve of MELD-XI predict no-reflow phenomenon. A sensitivity of 81.90% and a specificity of 66.67%. **b** ROC curve of MELD-XI predict 30-days all cause mortality. A sensitivity of 75.0% and a specificity of 64.5%

**Fig. 2:**
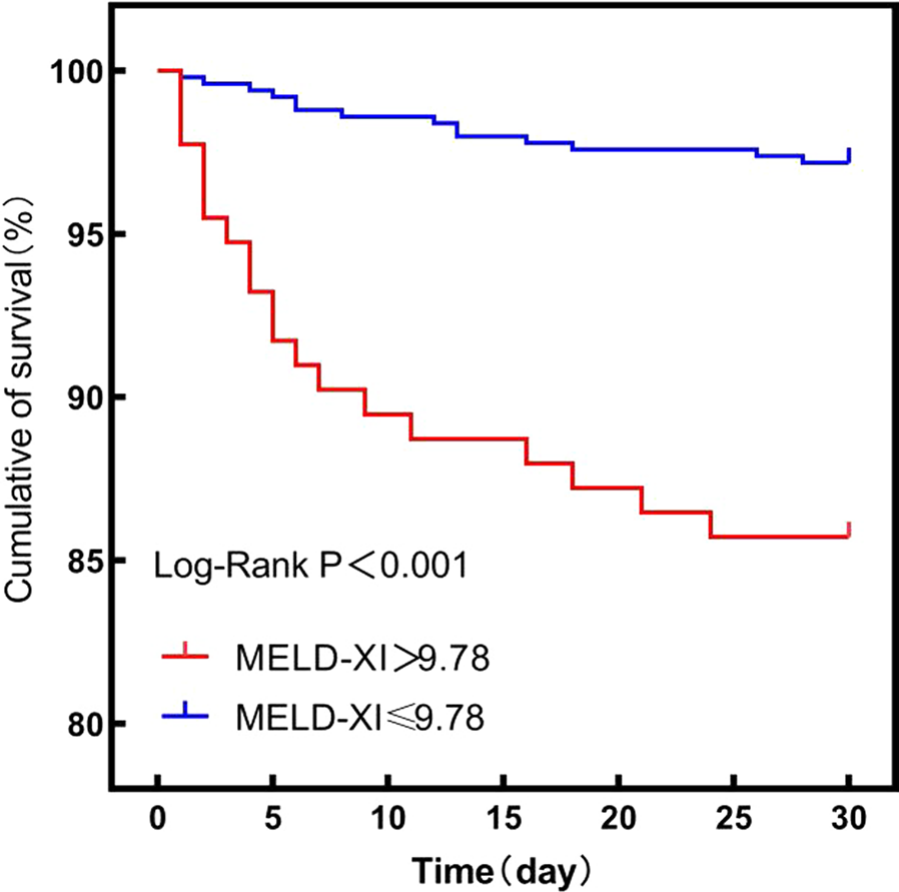
Kaplan–Meier curves of MELD-XI ≤ 9.78 and > 9.78 in patients with STEMI undergoing PPCI for 30-days all-cause mortality

The independent predictors of NRP are given in Table 2. Univariate and multivariate logistic regression analyses revealed that diabetes mellitus (odds ratio [OR]: 2.173, 95% CI: 1.423–3.227, *P* < 0.001), peripheral vascular disease history (OR: 3.448, 95% CI: 1.412–8.421, *P* = 0.007), MELD-XI scores (OR: 1.247, 95% CI: 1.144–1.360, *P* < 0.001), TB levels (OR: 1.046, 95% CI:1.023–1.070, *P* < 0.001), sCr (OR: 1.014, 95% CI: 1.007–1.021, *P* < 0.001) were significant and independent predictors of NRP. ROC curve analyses revealed that the optimal cut‐off value of MELD-XI score in predicting NRP was 9.47, with 81.90% sensitivity and 66.67% specificity (AUC = 0.739, 95% CI: 0.696–0.783, *P* < 0.001, Fig. 1a). Cox regression multivariate analyses showed that 30-day mortality, heart failure, hs-CRP, CK-MB, and the MELD-XI score upon admission were independent predictors (Table 3). Among all the patients, 36 (5.2%) died while admitted to the hospital and 30 day after they were discharged. ROC curve analyses revealed that MELD-XI scores of ≥ 9.78 had a sensitivity of 75.0% and a specificity of 64.5% (AUC: 0.742, 95% CI: 0.650–0.833, *P* < 0.001) (Fig. 1b). Kaplan–Meier curve analysis revealed that the patients with MELD-XI scores of > 9.78 group had worse outcomes in terms of 30-day all-cause mortality (*P* < 0.001) (Fig. 2).

**Table 2:** Logistic regression analyses for development of no-reflow phenomenon

Variable	Univariate regression		Multiple regression	
	OR (95% CI)	*P*	OR (95% CI)	*P*
Age (years)	1.010 (0.995–1.025)	0.181		
Male, n (%)	0.795 (0.499–1.265)	0.333		
Smoking, n (%)	0.691 (0.475–1.006)	0.054		
Heart failure (Killip II–IV, class), n (%)	1.774 (1.203–2.614)	0.004	1.381 (0.903–2.112)	0.137
Hypertension, n (%)	1.887 (1.272–2.797)	0.002	1.432 (0.936–2.191)	0.098
Diabetes mellitus, n (%)	2.256 (1.555–3.272)	< 0.001	2.173 (1.463–3.227)	< 0.001
Stroke or TIA, n (%)	1.067 (0.583–1.954)	0.833		
Family history, n (%)	1.023 (0.407–2.571)	0.962		
Peripheral vascular history, n (%)	5.168 (2.294–11.644)	< 0.001	3.448 (1.412–8.421)	0.007
Hyperlipidaemia, n (%)	0.739 (0.512–1.068)	0.107		
SBP, mmHg	1.009 (1.001–1.017)	0.031	1.007 (0.999–1.016)	0.100
LVEF (%)	0.990 (0.972–1.010)	0.327		
TC (mmol/L)	1.052 (0.889–1.245)	0.556		
TG (mmol/L)	0.889 (0.758–1.041)	0.144		
hs‐CRP (mg/L)	1.004 (1.000–1.008)	0.052		
CK-MB (U/L)	1.000 (0.999–1.001)	0.939		
UA (μmol/L)	1.001 (0.999–1.002)	0.464		
MELD-XI score	1.276 (1.176–1.385)	< 0.001	1.247 (1.144–1.360)	< 0.001

*OR* odds ratio, *CI* confidence interval

**Table 3:** Cox regression analyses revealing the predictors of 30-days all-cause mortality in patients with STEMI undergoing PPCI

Variable	Univariate regression		Multiple regression	
	HR (95% CI)	*P*	HR (95% CI)	*P*
Age (years)	1.039 (1.010–1.069)	0.009	1.008 (0.978–1.039)	0.606
Male, n (%)	0.617 (0.290–1.311)	0.209		
Smoking, n (%)	0.731 (0.377–1.417)	0.353		
Heart failure (Killip II–IV, class), n (%)	3.866 (1.993–7.500)	< 0.001	2.270 (1.017–5.066)	0.045
Hypertension, n (%)	2.936 (1.286–6.703)	0.011	2.144 (0.908–5.063)	0.082
Diabetes mellitus, n (%)	0.712 (0.344–1.477)	0.362		
Stroke or TIA, n (%)	1.147 (0.405–3.242)	0.796		
Family history, n (%)	0.670 (0.092–4.894)	0.693		
Hyperlipidaemia, n (%)	1.111 (0.569–2.172)	0.758		
Heart rate (bpm)	1.010 (0.989–1.031)	0.360		
SBP, mmHg	0.997 (0.983–1.012)	0.732		
LVEF (%)	0.954 (0.922–0.987)	0.006	0.980 (0.946–1.016)	0.274
hs‐CRP (mg/L)	1.010 (1.005–1.015)	< 0.001	1.005 (1.000–1.011)	0.036
CK-MB (U/L)	1.002 (1.001–1.004)	0.001	1.002 (1.001–1.003)	0.005
UA (μmol/L)	1.002 (0.999–1.005)	0.190		
Multivessel vessel, n (%)	2.027 (0.924–4.447)	0.078		
MELD-XI score	1.208 (1.140–1.280)	< 0.001	1.155 (1.077–1.239)	< 0.001

*HR* hazard ratio, *CI* confidence interval

## Discussion

This study confirmed that patients with NRP had substantially higher MELD-XI scores as evaluated upon admission than patients with normal reflow. Moreover, this study established that the MELD-XI score is an important independent predictor of NRP in patients with STEMI undergoing PPCI. Furthermore, this study verified that the MELD-XI score has a strong predictive power for 30-day all-cause mortality in patients with STEMI after PPCI. This study was the first to evaluate the utility of the MELD-XI score in the risk stratification of NRP and 30-day all-cause mortality in patients with STEMI after PPCI.

Multiple factors contribute to the occurrence of NRP, but the exact mechanism is complex and remains unclear. The main mechanisms proposed are spasm or obstruction of microcirculation, distal microvascular embolization, long ischemic duration, platelet aggregation, oxidative stress, and ischemic or reperfusion injury [11]. NRP remains a powerful independent predictor of early and long-term death in patients with STEMI. Patients who eventually develop NRP experience MACEs and complications more frequently than those with normal reflow [12]. Therefore, NRP and its short-term risks should assessed among patients who are candidates to undergo PCI to improve the safety of the intervention.

Several available biomarkers and some easily obtained clinical parameters have been demonstrated to be able to predict NRP [13, 14]. Bilirubin is the main bile pigment in human bile and a natural metabolized product of iron porphyrin compounds. Oxidized low-density lipoprotein-induced reactive oxygen species (ROS) production is a harmful cholesterol and a risk factor for atherosclerosis [15, 16]. Several studies have demonstrated that bilirubin is a potent endogenous antioxidant than can suppress ROS. Thus, bilirubin can inhibit the oxidation process of LDL and plays an important role in preventing the progression of atherosclerosis via its antioxidant activity. Endothelial dysfunction may be caused by the ROS produced by oxidative stress [17]. Previous studies have illustrated that endothelial cell damage caused by excessive oxidative stress has a strong association with NRP in patients with STEMI [18–20]. Celik et al. [4] examined the associations between TB levels and NRP and in-hospital MACEs. They found a tight association between high bilirubin levels with low TIMI flow grades and NRP in patients with STEMI [4]. This finding suggests that bilirubin is key mediator in inhibiting inflammatory processes, especially those accompanied by oxidative stress.

Creatinine is the anhydride form of creatine. It has been proposed as a marker of renal function [21]. Functional changes in the kidneys can be monitored by estimating serum creatinine. High serum creatinine upon admission is a risk factor of NRP [22]. Mild to moderate renal impairment in patients with STEMI who had undergone PPCI is independently associated with NRP [23]. The exact mechanisms of renal dysfunction and NRP include the accumulation of elevated ROS, inflammatory process, and endothelial damage [24]. Endothelial dysfunction is strongly associated with renal dysfunction and impaired myocardial perfusion in patients with STEMI [25]. NRP is partly a result of inflammation-induced reperfusion injury. Several studies have established that renal dysfunction is also an inflammatory state, and NRP is associated with inflammatory activity [26–28]. A certain correlation exists between oxidative stress and NRP as confirmed by a previous study [19]. The generation of oxidative stress also plays a crucial role in renal impairment [29]. All these conditions are probably involved in the development of NRP. In the present study, the patients in the no-reflow group had higher values of C-reactive protein than those in the normal flow group after PCI (12.10 vs. 8.13, respectively; *P* = 0.017). In a previous study, inflammation markers have been shown to be associated with NRP development in patients with STEMI treated with PPCI [30]. Among these inflammation markers, CRP may mediate complementary activation and neutrophil plugging, causing microvascular damage to the development of NRP [31]. Furthermore, CRP is one of the common biomarkers associated with mortality in patients with acute coronary syndromes [30]. The present study also showed that CRP levels in patients with STEMI who underwent PPCI are an independent factor for 30-day all-cause mortality, in accordance with the results of previous studies.

Several studies have recently shown that elevated TB has a good predictive value in the prognosis of patients with acute coronary syndrome who had undergone PCI [32, 33]. Impaired renal function plays an important role in predicting the future occurrence of MACEs in patients with PCI [34]. Vinod et al. [35] reported that patients who have elevated serum creatinine upon admission are accompanied with increased risk of developing MACEs.

The MELD-XI score is a novel and easily accessible scoring system. It only requires two parameters, namely, TB and sCr, which can be determined through noninvasive blood test. As a tool for assessing liver and kidney functions, the MELD-XI score not only reflects the critical condition of patients with organ failure but also closely estimates in-hospital mortality. A previous study demonstrated that the MELD-XI score shows good predictive ability for in-hospital and 1-year mortality in older patients with STEMI who had undergone PCI [36]. However, the authors did not explore whether their conclusion could be extrapolated to other age strata. Our results showed that patients with a high MELD-XI score have a high rate of short-term all-cause mortality. Moreover, multivariate Cox regression analysis revealed that the MELD-XI score, CK-MB, and hs-CRP upon admission are independent predictors of short-term prognosis. Renal and hepatic functions may have a high predictive power for the mortality of patients with cardiovascular diseases. Patients with NRP have the highest risk of early and congestive heart failure and death. This study consistently found that NRP is associated with poor prognosis and high mortality rate of patients with STEMI after PPCI. The MELD-XI score showed a high predictive power for coronary NRP and short-term prognosis. Thus, it can be used in the early risk stratification of patients with STEMI who are candidates for PPCI.

## Limitations

Several limitations should be considered in our study. First, this is a retrospective single-center study, and sample size was relatively small, bias could not be completely ruled out. Therefore, these conclusions should be verifed in in different regions and different populations with larger samples. Second only end-stage liver disease or on dialysis were excluded. not all patients with previously diagnosed liver disease and renal disease.

## Conclusion

This study indicated that MELD-XI score was associated independently and signifcantly with NRP and 30-days prognosis in STEMI patients undergoing PPCI. MELD-XI may be applied as a early risk stratification tools of recognizing high-risk patients to improve their clinical outcomes.

## Data Availability

The database used and/or analyzed for this study available from the corresponding author on reasonable request (Liang-Long Chen; Email: lianglongchenxh@126.com).

## Abbreviations

MELD-XI: The model for end-stage liver disease excluding international normalized ratio
NRP: No-reflow phenomenon
TIMI: Thrombolysis in myocardial infarction
STEMI: ST-segment elevation myocardial infarction
ACS: Acute coronary syndrome
PPCI: Primary percutaneous coronary intervention
ROC: Receiver operator characteristic
MACEs: Major adverse clinical events
AUC: Area under the curve
TC: Total cholesterol
TG: Triglycerides
LDL-C: Low density lipoprotein cholesterol
oxLDL: Oxidized low density lipoprotein
HDL-C: High density lipoprotein cholesterol
UA: Uric acid
BUN: Blood urea nitrogen
sCr: Serum creatine
TB: Total bilirubin
Nt-proBNP: N-terminal pro-brain natriuretic peptide
CK-MB: Creatine Kinase-MB
hs-CRP: High sensitivity C-reactive protein
LVEF: Left ventricular ejection fraction
SBP: Systolic blood pressure
DBP: Diastolic blood pressure
OR: Odds ratio
HR: Hazard ratio
CI: Confidence interval
LMCA: Left main coronary artery
